# Supplementary material for: Are carbon emissions trading and green financial instruments synergistic? -Comprehensive quantitative research based on content analysis
Source: PLoS One. 2024 Mar 7;19(3):e0298601. doi: 10.1371/journal.pone.0298601 (PMC10919601; doi:10.1371/journal.pone.0298601)
Supplement: S1 Data — (ZIP) [file pone.0298601.s001.zip › Data/地级市Z_F工作报告.zip!/╣π╢1⁄2/│▒╓▌╩╨/└┤╫╘╣1⁄2╓┌║┼í╛┬φ┐╦╩2╛▌═°í┐_2019.docx]

各位代表：

现在，我代表市人民政府向大会作工作报告，请予审议，并请市政协委员和其他列席人员提出意见。

2018年工作回顾

过去一年，市政府在省委、省政府和市委的正确领导下，在市人大和市政协的监督支持下，坚持以习近平新时代中国特色社会主义思想为指导，全面贯彻党的十九大和十九届二中、三中全会精神，深入贯彻习近平总书记对广东重要讲话和重要指示精神，围绕我省实现“四个走在全国前列”、当好“两个重要窗口”，按照省委“1+1+9”的工作部署和李希书记莅潮调研时关于“打造沿海经济带上的特色精品城市”的发展定位，认真落实省委、市委全会的决策部署，坚持稳中求进总基调，坚持新发展理念，按照高质量发展的要求，全面深化供给侧结构性改革，打好三大攻坚战，统筹做好稳增长、促改革、调结构、惠民生、防风险各项工作，全力以赴稳就业、稳金融、稳外贸、稳外资、稳投资、稳预期，经济社会各项事业加快向高质量发展阶段迈进。预计全市实现生产总值1090亿元，增长5.5%；人均生产总值4.1万元，增长4.85%；一般公共预算收入47.4亿元，增长6.2%；各项税收总收入100.9亿元，增长5.3%；固定资产投资424.8亿元，增长3.4%；外贸进出口总额211.9亿元，增长0.5%；社会消费品零售总额587.6亿元，增长8.8%；城乡居民人均可支配收入增长7.9%；居民消费价格指数涨幅控制在3%以内。

一年来，我们坚持以高质量发展为主题，着力建设现代化经济体系，产业质效进一步提升。一是经济实力持续壮大。全面落实省“实体经济十条”“民营经济十条”“金融十条”，制订实施“1+N”产业扶持政策和《潮州市推动陶瓷产业高质量发展实施方案》等扶企惠企政策，减免各项税费7.5亿元。上市公司、新三板挂牌企业、区域性市场挂牌企业增至55家。规上工业企业增至923家；产值超亿元企业增至344家；完成“个转企”1072家，增长88.2%；新认定“四梁八柱”企业56家；“广东老字号”增至26家，数量居全省第2位；我市获评“中国食品名城”。二是新兴动能显著增强。先进制造业占规上工业比重增至20.2%。凤泉湖高新区、樟溪低碳工业园等产业园区建设竞相推进，完成征地3549.9亩，建成工业地产6.7万平方米，年度累计投资达7.2亿元。“一园六区”新引进项目32个，超亿元项目17个，益海嘉里等一批百亿级项目落地建设，雅士利、恒洁等一批本土企业回归。三是科技创新成果丰硕。全面推进科技创新“十大工程”，市级科技专项资金投入翻番。全国唯一的国家级餐具炊具知识产权快速维权中心开通运营，外观设计专利审查、授权时间缩短80%以上。先进陶瓷材料创新研究中心建成投用，化学与精细化工广东省实验室潮州分中心启动建设。高新技术企业突破100家，增长43%，通过率居全省首位。新型研发机构增长一倍，新增省级工程中心6家、省级企业技术中心3家。每万人发明专利拥有量居粤东西北各市第2位。三环公司获省科学技术奖一等奖。四是产业结构有效优化。三次产业结构调整优化为6.9：49.1：44。饶平县现代农业（茶叶）产业园列入2018年省级首批建设名单，全市农业合作社达935家。“潮州老香黄”“潮州手拉朱泥壶”获评国家地理标志产品。饶平县水产品质量安全示范区被认定为“省级出口食品农产品质量安全示范区”，三百门国家一级渔港通过农业部验收，北部万亩淡水养殖示范区加快建设。电子商务发展迅猛，饶平县获评全国“电商示范百佳县”。“全域旅游示范区”创建工作全面推进，我市获评“最喜爱国内游目的地”，潮安区被授予“中国十佳特色文化旅游名区”，凤凰茶旅小镇和太安堂医养小镇入选省级特色小镇创建名单。全市接待海内外游客2007万人次，实现旅游收入306亿元，分别增长31%和30%。

一年来，我们坚持以重点项目为抓手，着力加快基础设施建设，发展根基进一步夯实。实施“百日攻坚”，124个市级重点项目完成投资186.6亿元。一是市政建设成效突出。“一江两岸”夜景提升工程获评“2018年阿拉丁神灯奖”。“八个十”项目有力推进，总投资6.5亿元的市城区40条道路改造提升工程已完工通车36条，一批小公园、停车场、肉菜市场改造项目顺利实施，“厕所革命”加快推进，绿榕南路、外环北路、东兴北路等一批步道完成改造和绿化提升。二是交通建设加速推进。完成投资44.6亿元，梅汕客专、护堤路枫溪段等项目征拆工作全面完成，广梅汕增建二线潮州段及厦深联络线顺利通车，潮汕站站改扩容工程全面推进，大埔至潮州港疏港铁路完成预可研报告并通过专家评审。高速公路建设施工里程达74.7公里，大潮高速和潮汕环线高速分别完成工程量的46.8%和48.5%，宁莞高速潮州东联络线前期工作顺利推进。潮州港扩建货运码头项目即将完工，公用航道一期工程顺利推进，金狮湾港区规划获省批准。我市成为全省5个“四好农村路”先行建设试点市之一。三是民生项目全面发力。总投资6.7亿元的13个公办学校项目扎实推进，市绵德小学、市绵德幼儿园动工建设，韩山师院附属实验学校完成主体工程。总投资24.3亿元的12个医院项目加快实施，市中医医院颐养院区投入使用，市中心医院新院、市妇幼保健院扩建工程等有效推进。完成村卫生站公建规范化建设396所，进度居全省首位。市档案馆新馆竣工投用，市图书馆新馆建成开放并晋升为国家一级馆。电网建设力度持续加大，年度完成投资10亿元。四是水利建设进展顺利。南堤和北堤综合整治工程全面完工，引韩济饶供水工程饶平段基本完成征地工作，韩江新城防洪综合整治工程（一期）、中小河流治理工程（二期）、凤城生态水乡水利整治等加快推进。

一年来，我们坚持以改革开放为重点，着力推进改革攻坚和开放合作，发展活力进一步激发。一是各项改革纵深推进。“放管服”改革成效明显，“绿色通道”列入事项157项，提速率达50.1%，开办企业和不动产交易登记办结时间分别压缩至4.5和5个工作日以内，工程建设项目审批时间压缩一半以上，整体通关时间压缩1/3。农信社改制顺利推进，我市获国务院批准为全国唯一市级统一法人农商行顺利申筹，成功压降不良贷款15.4亿元，网点资产确权发证197宗，金融风险得到有效防控。融资服务力度加大，依托中小微企业贷款风险补偿基金、转贷风险补偿基金等平台，累计引导促成1682家企业融资近120亿元。国税地税征管体制改革稳妥推进，市县镇三级税务机构完成合并挂牌。执法机制改革加紧推进，“一单两库一细则”“双随机一公开”监管模式实现全覆盖，清理无照经营并核发营业执照1.6万多户。“信用潮州”系统完成数据归集超91万条，实现全市信用系统区域全覆盖和全省信用数据“一网通查”，541家企业被授予省“守合同重信用企业”称号。强化企业信用约束和失信惩戒，企业年报率在全省排名第6位。消费维权扎实推进，“投诉处理回复率”和“消费投诉处理率”均列全省排名第1位，“行政维权”工作全省排名第2位。农电体制改革稳步推进，意溪供电有限公司产权顺利划转。交通运输改革全面深化，实施公交行业、人力三轮车等5项改革措施，系统重构公共交通运输服务体系。二是人才机制完善优化。深入落实“人才新政23条”，发布首个急需紧缺人才目录，建立人才工作香港、上海联络站，创新人才驿站、人才咖啡等服务模式。创新事业编制周转池制度，设立人才储备中心，核定高层次人才储备专项事业编制100名。新引进博士69名、硕士254名，新增博士后科研工作站1个、博士工作站12个，30个项目入选省“扬帆计划”。三是对外开放全面拓展。积极参与“一带一路”建设，组织企业参加广交会、上海尚品家居展、进博会等展会。成功举办2018潮州国际刺绣艺术双年展、“世界潮文化发祥地·潮州”全国摄影大赛、“等你千年·爱在潮州”婚恋旅拍活动，申获“潮州陶瓷全球采购基地”，再次获评“中国卫生陶瓷出口基地”。制定实施“外资十条”，全市新增外资项目68个，实际利用外资增长43.7%。四是区域合作不断深化。做实做深与中山市对口帮扶合作，产业共建稳步推进，潮州青年文创中心建成投用，“中潮慈善爱心基金”已发放善款489万元。加强市际交流合作，推动与广西柳州缔结为友好城市，汕潮揭同城化有序推进。加强文化交流，先后在法国巴黎、意大利米兰、泰国曼谷等地举行“潮州文化交流周”“中国精品刺绣艺术展”等活动，柬埔寨、马来西亚、菲律宾等地“潮文化海外交流中心”挂牌成立。

一年来，我们坚持以综合治理为突破，着力补齐短板弱项，发展环境进一步优化。一是城乡环境全面升级。新一轮城市总体规划获省批准，地下管廊、海绵城市等专项规划编制完成，《潮州市城乡居民住宅建设管理办法》制定实施。智慧交通、智能停车收费等系统投入使用，城市管理智能化水平逐步提高。常态化开展“治六乱”工作，完成“两违”清拆655宗78万平方米，收缴无牌无证摩托车3.8万多辆。二是平安创建全面强化。实现刑拘、逮捕、起诉人员“三上升”和刑事警情、治安警情、“两抢一盗”警情“三下降”，社会大局持续稳定。扫黑除恶专项斗争深入开展，破获涉黑恶案件356宗，打掉涉黑恶犯罪团伙94个，查处“保护伞”案件22宗，涉案资金财物1.6亿元。电信诈骗专项整治成效突出，在逃人员全部清零。推广基层治理“四化”建设试点经验，社会治安共建共治共享格局更加完善。加强矛盾纠纷多元化调解机制建设，建立健全“以案定补”和聘请专职人民调解员工作制度，实现到省进京非正常访“零发生”，信访总量全省最低。积极申报创建全国禁毒示范市，应急救援等工作扎实开展。推进“雪亮工程”“视频云”工程建设，建成“归一大数据平台2.0”，智能执法魔方等落地使用，提前超额实现全警100%配置警务终端硬目标，移动警务本地化应用领跑粤东。严格落实安全管理“一岗双责”“三个抓”，完成17个“城中村”消防安全整治，地质灾害监测防治、安全生产监管等专项工作扎实有效。强化食品药品安全防控，30家农贸市场实施食用农产品快检。三是环保整治全面深化。坚决落实中央环保督察及“回头看”整改要求，划定并严守生态保护红线，实施“双随机一公开”综合执法监管。完成饮用水源保护区优化调整工作，推进饮用水水源地环境问题整治，县级以上饮用水源达标率100%。全面开展城市空气质量达标管理，推动扬尘污染防治立法，空气质量居全省第10位。推进环保设施补短板工作，枫江流域一期整治工程全面完工，市第一污水处理厂提标扩容工程投入使用，市第二污水处理厂配套主干管和污泥处理中心一期工程、桥东污水处理厂提标工程等建成。深入落实河长制、湖长制，组织开展“碧水清流·共建共享”万人大巡河活动，构建生态环境监测网络，重点河流断面水质自动监测站一期工程建成并联网运行。西澳岛生态修复项目主体工程完工。创建“国家森林城市”，新增森林碳汇造林1.1万亩、森林抚育14.6万亩、桉树林改造2.1万亩。

一年来，我们坚持以人民为中心的发展思想，着力推动共建共治共享，民生福祉进一步增强。全市民生领域投入资金145.8亿元，占一般公共预算支出78.8%。一是社会保障日臻完善。新增城镇就业1.5万人，城镇登记失业率控制在3.5%以内。城乡居民基本养老、基本医疗保险参保人数达到102.3万人和230.8万人，大病保险减轻参保人负担1.2亿元。低保、特困、孤儿、残疾人等底线民生保障水平有效提升。住房租赁补贴完成省下达任务的225%。二是公共事业蓬勃发展。教育现代化先进市创建步伐加快，已投入资金16.7亿元，实现“广东省推进教育现代化先进县（区）”全覆盖。组建联盟化办学共同体，成立韩山师范学院实验学校教育集团。实施公立医院综合改革，多形式医联体建设成效明显。大力繁荣文化事业，村级综合文化服务中心实现全覆盖。成功举办省民歌民乐大赛和首届潮州市骑行节等文体活动，在央视《春节戏曲晚会》《唱响新时代》《改革开放四十周年》等栏目推出潮州专题节目。新增国家级非遗传承人6名、省级非遗项目4个，我市被授予全国唯一一个“中国民间工艺传承之都”。对涵碧楼、茂芝会议旧址等9个红色革命遗址和红色纪念场馆进行修复保护利用，建成一批红色文化讲习所。扶持推动民办博物馆蓬勃发展，实施“民办公助”模式，形成“国有博物馆为龙头、行业博物馆为骨干、民营和私营博物馆为主体”的博物馆体系，51家民间博物馆获得授牌。实施古城保育活化工程，创新“公办民助、私建公管”模式，推进“百家修百厝（祠）”，海阳县儒学宫、李厝祠等文物点得到更好保育，西马路、义安路、美食街建设顺利推进，古城区标准化旅游导览标识系统全面建成。三是脱贫攻坚精准发力。建成农业特色产业扶贫示范基地超1.3万亩，获评全省“十大产业扶贫典型案例”。精准扶贫“村村通就业信息网”实现镇村全覆盖。有劳动能力的贫困户人均可支配收入达到9586元，新增预脱贫0.87万人，完成农村危房改造610户，贫困人口城乡基本医疗保险、养老保险和低保等兜底保障工作有效落实，精准扶贫工作取得阶段性成效。落实贫困村创建新农村示范村资金5.4亿元，贫困村通村道路硬底化完成率100%。四是乡村振兴卓有成效。编制实施《潮州市美丽乡村民居建筑设计指引》等文件，统筹安排1亿元资金启动250个行政村美丽乡村建设，新农村连片示范建设工程扎实推进。456个行政村全部完成村庄初步规划编制，覆盖率居全省第3位，农村土地确权工作基本完成。大力实施“潮州菜师傅”工程，成功举办潮州菜名师、名店、名菜评选活动，“送教上门”培养乡村厨师400多名，形成“一村一店一菜”乡村产业化项目。与此同时，人事、司法行政、民族宗教、外事侨务、港澳、对台、审计、统计、贸促、物价、粮食、打私、打假、人防、气象、防震、地方志、档案、拥军优抚、民兵预备役、老龄、妇女儿童等工作取得新成绩。

一年来，我们坚持以党建为引领，着力加强行政能力建设，政府效能进一步提升。一是体制机制更加完善。认真落实新时代党的建设总要求，深入贯彻落实习近平总书记重要讲话精神，探索制订高质量发展等制度机制和评价体系，政府工作的导向力和推动力全面增强。二是行政服务更加高效。加快“数字政府”建设，打造“一网通办”平台，完成政务服务平台集约化改造提升。编制《潮州市市级公共服务事项清单》，梳理市级公共服务事项352项。实施政务信息系统整合共享，政府数据开放数量居全省前列。三是民主法治更加健全。全面贯彻实施宪法，自觉接受人大、政协和社会各界监督，办结人大代表建议27件、政协委员提案106件。推进法治政府建设，出台实施全国首部地级市古城消防管理政府规章《潮州市古城区域消防安全管理办法》，重大行政决策合法性审查、社会听证等制度有效落实，行政决策更加科学民主。四是廉政建设更加深入。建立预决算信息公开的常态化机制，全面推行零基预算，建立专项资金项目库，严格控制预算追加。严控“三公”经费，党政机关单位会议费及“三公”经费财政拨款支出下降10.9%。全面落实党风廉政建设责任制，深入推进反腐倡廉，强化审计监督，重大政策贯彻落实情况跟踪审计有效开展。

各位代表，过去一年的成绩来之不易。这是省委、省政府和市委坚强领导的结果,是全市人民团结拼搏、苦干实干的结果，是市人大、市政协监督支持的结果，也是驻潮部队、武警官兵、上级驻潮各单位和海内外各界人士鼎力相助的结果。在此，我代表市人民政府表示衷心的感谢并致以崇高的敬意！

与此同时，我们也清醒地认识到，我市经济社会发展仍存在一些困难和问题，主要是：实体经济发展的外部环境更趋复杂严峻，部分企业生产经营困难；经济发展质量和效益还需进一步提高，创新能力有待增强，高端人才紧缺，基础研究和原始创新短板突出，新兴产业还不能挑起大梁，发展新动能仍然不足；城乡区域发展不平衡不协调，城市整体发展水平与珠三角城市差距大，农村地区内生发展动力不强，二元结构问题突出，基础设施配套不完善，各大平台的辐射带动效应尚未充分显现；公共服务资源供给能力与人民群众日益增长的美好生活需要还存在差距，教育、医疗卫生、文化、体育等公共服务供给不足、质量不高，保障和改善民生的能力水平仍需不断提高；一些领域生态环境问题依然突出，绿色生产生活方式尚未形成；高质量发展体制机制不够健全，改革攻坚还需持续深入推进，法治建设有待进一步加强。对此，我们一定高度重视，采取强有力措施加以解决。

2019年工作安排

2019年是新中国成立70周年，是全面建成小康社会关键之年。当前，国际经济合作和竞争格局发生深刻变化，保护主义、单边主义明显抬头，经济运行稳中有变，外部环境复杂严峻，经济面临下行压力。随着我国经济需求结构快速调整，消费结构全面升级，对提升供给质量、优化产业布局提出了新的更高要求。与此同时，我们面临的利好因素正在不断叠加，国家“一带一路”倡议和深化改革开放的红利逐步释放，省“一核一带一区”发展战略深入实施，市“一体两翼三带四平台”建设全面推进，为潮州加快发展带来难得的机遇和强大的动力。我们要保持战略定力，抢抓发展机遇，进一步解放思想、深化改革、扩大开放，在更高起点、更高层次、更高目标上推动潮州经济社会实现高质量发展。

今年政府工作的总体要求是：高举习近平新时代中国特色社会主义思想伟大旗帜，全面贯彻党的十九大和十九届二中、三中全会以及中央经济工作会议精神，全面贯彻落实习近平总书记对广东重要讲话和重要指示精神，认真贯彻落实省委十二届六次全会和市委十四届七次全会精神，围绕打造“沿海经济带上的特色精品城市”发展目标，统筹推进“五位一体”总体布局，协调推进“四个全面”战略布局，坚持稳中求进工作总基调，坚持新发展理念，坚持推动高质量发展，坚持以供给侧结构性改革为主线，坚持深化改革扩大开放，统筹做好稳增长、促改革、调结构、惠民生、防风险、保稳定各项工作，进一步稳就业、稳金融、稳外贸、稳外资、稳投资、稳预期，提振市场信心，打好三大攻坚战，着力提高发展平衡性和协调性，坚持不懈推进全面从严治党，保持经济持续健康发展和社会大局稳定，为全面建成小康社会打下坚实基础，以优异成绩庆祝中华人民共和国成立70周年。

综合各方面因素，今年我市经济社会发展的主要预期目标是：生产总值增长6-6.5%，一般公共预算收入增长6%，固定资产投资增长8%，外贸进出口总额增长1%，全社会消费品零售总额增长8.5%，居民消费价格指数涨幅控制在3%以内，居民收入增长和经济增长基本同步，城镇登记失业率控制在3.5%以下、调查失业率控制在5%以内。

为实现上述目标，我们将重点做好以下五方面工作。

一、推动高质量发展，构建现代产业新体系

把发展经济的着力点放在实体经济上，深入推进供给侧结构性改革，全力推动经济发展质量变革、效率变革、动力变革。

深化服务，全面创优营商环境。深入落实习近平总书记在民营企业座谈会上的重要讲话精神，贯彻实施“实体经济十条”“民营经济十条”等系列惠企措施，落实领导挂钩服务企业制度，以清单式、责任制形式为企业解决实际困难，大力营造国际化法治化便利化营商环境，激发微观主体活力，扶持壮大实体经济。着力破解“成本高”问题，落实减税降负政策，推行“直购电”模式，推进燃气“一张网”建设，切实降低企业税费和用电用气等成本。着力破解“融资难”问题，发挥中小微企业贷款风险补偿基金、中征应收账款融资服务平台等的积极作用，促成银企“无缝对接”。着力破解“招工难”问题，建立覆盖全市农村的招用工平台，加强校企对接合作，采用“订单式”精准培养技能型人才。着力破解“审批难”问题，深入落实“三集中一分离”运行机制和“绿色通道”服务模式，健全容缺受理、代办专班等服务制度，探索实施多评合一、多图联审、联验联审、电子勘探等新模式、新措施，将企业开办和项目审批时间压减一半。

狠抓创新，加快建立技术创新体系。坚持创新是第一动力，深入实施科技创新“十大工程”。加快推动广东省实验室潮州分中心建设，推动陶瓷材料、食品科学与技术两个领域的应用基础研究。整合韩山师院、三环集团和陶研机构等科研资源，实施“柔性合作”“柔性引才”机制，集中“优势兵力”推动高性能陶瓷等共性关键技术攻关突破，打通产学研用链条，全力抢占全球陶瓷产业制高点。发挥企业创新主体作用，实现规模以上工业企业建立研发机构比例提高到35%。深入实施“互联网+”行动计划，推进国家、省智能制造示范项目和“机器人应用”项目。以三环先进材料生产基地、智能高档卫浴洁具生产总部基地等项目为带动，大力推广数控机床、工业机器手等先进设备，打造一批数字化车间、智能工厂，推动制造业加速向数字化、智能化发展。整合力量创建国家级高新区和创新驱动先行区，启动建设中山-潮州（潮安）产业创新中心，加快推进广东潮汕高教科技研究院建设，规划建设科技园区。加强高新技术企业培育，力争高企数量突破130家。创建国家知识产权示范城市，健全知识产权维权保护体系。

大力提质，积极发展实体经济。坚持传统产业与新兴产业并重并推，下足功夫提高制造业的质量和效益，推进“个转企”“小升规”等工作，做好“四梁八柱”企业培育认定工作。致力推动传统产业提质升级，以陶瓷、食品、服装等8大支柱产业和21个省级专业镇为重点，深入实施新一轮工业企业技术改造。加大“中国瓷都”“中国食品名城”等金字招牌的宣传推介力度，推进陶瓷原材料升级、智能制造、价格指数编制等六大方面工作，力促陶瓷、食品等传统产业加快高质量发展。致力推动新兴产业发展壮大，实施《潮州市优先发展产业目录》，大力引进一批投资规模大、技术水平高、产业带动强的战略性新兴产业，推动益海嘉里以及太安堂医养小镇等重大产业项目建设投产，全力打造新材料、新能源、生物医药健康等“5个500亿级”产业集群。推进质量强市建设，开展质量提升行动。深入实施标准化战略，推进潮州菜、潮州工夫茶等团体标准建设。

构筑平台，深度参与沿海经济带建设。抢抓省建设“一核一带一区”战略机遇，高起点制订我市参与沿海经济带建设总体规划，构建大平台，狠抓大项目，着力建设沿海沿江沿高铁经济带。重点打造凤泉湖高新区、闽粤经济合作区、高铁新城、韩江新城等四大发展平台，谋划实施澄饶联围综合整治和规划建设，加快推进临港产业园、樟溪低碳工业园、东山湖现代产业园、古巷产业园、官塘工业园等园区开发建设。突出园区基础设施建设攻坚，抓好园区“七通一平”，加快推动潮州港扩建货运码头等交通设施，以及污水处理、天然气管网、公租房、公共交通等生产生活配套建设，全面增强园区发展承载力。扶持工业地产发展，推动通用厂房建设投用。坚持以大项目带动大发展，创新招商引资模式和产业共建模式，抢抓国家对新上重大项目能耗指标实行单列的政策机遇，大力实施精准招商，建立重大项目洽谈引进“绿色通道”，千方百计引进推动一批重大产业项目落地建设。抓好入园落地项目的促产增效，力促一批项目投产量产、创造效益。建设总部企业基地，推进总部企业引进认定工作，培育形成总部经济业态。

发展商贸，做活做大第三产业。培育发展文旅商贸、科技信息等现代服务业，探索推动先进制造业与现代服务业深度融合。推动一批商贸综合体和高端酒店建设，培育形成中高端消费业态。深入推进“信用消费进万家”，瞄准国家促进形成强大国内市场的目标导向，开展扩内销促消费活动，不断壮大消费市场。扶持发展电子商务，实施电商人才培育工程，助力农村电商发展。大力发展文化旅游产业，加快创建“全域旅游示范区”，推进“品味潮州”品牌建设，以“绣花”功夫推进南门十巷、义安路、西马路以及“百家修百厝（祠）”等古城微改造项目，保护开发笔架山潮州窑考古遗址公园和古城博物馆群，推动美食街、手信街、老字号展示馆等建设，推广“潮州人家”民宿品牌，努力让古城留下记忆，让天下潮人记住乡愁。引进国内顶尖文旅管理机构，以整合优化思维推进古城建设运营，统筹好集散中心、停车场以及“智慧旅游”系统等配套建设，全面提升古城旅游品位层级。大力发展乡村旅游，加快打造一批“古镇古寨古村”，拓展工业旅游、红色旅游、滨海旅游。大力发展文创产业，推进中国工艺美术之都博览园、潮州文化金融街等项目，提升中山路工艺美术一条街，推动木雕、麦秆画等传统工艺工业化、产业化、规模化，促进文商旅深度融合。

二、深化改革开放，抢抓区域发展新机遇

把握好粤港澳大湾区建设战略机遇，全方位推动对接融入和开放合作，深入推进体制机制改革，全面提升改革开放的质量和水平。

在更广范围上融入粤港澳大湾区。全力做好“融”字文章，充分发挥高铁站、机场、高速公路和港口等叠加形成的交通枢纽优势，深度融入大湾区综合交通体系，加大对接拓展延伸力度。推动进港公路延长线、滨海旅游公路建设，加快疏港铁路前期工作，加大潮州港与广州港合作力度。深化与大湾区的产业对接合作和经贸交流，探索优化招商产业链上下游互补机制，扩大招商引资规模，积极承接产业转移。主动学习借鉴广东自贸区创新经验，全面推广国际贸易“单一窗口”。加强与中山市的对口帮扶合作，推进产业项目、人才驿站和创新平台建设。结合汕潮揭同城化，推动临港空铁经济合作区发展总体规划落地实施，扶持发展空铁港区的现代物流、后勤保障、生活服务等配套产业，规划建设高铁潮汕站区商贸物流中心、潮州特色产品物流区。

在更高水平上扩大开放。积极参与“一带一路”建设，组织企业参加国内外各类展会，通过设立“中国瓷都·潮州”“中国·潮州食品名城”等专馆、专区的形式，全力开拓市场空间。着力优化提升外贸业态，发挥“国家农业对外开放合作试验区”等平台作用，推进“新外贸示范市”建设，设立新外贸本地化服务中心。积极应对严峻外贸形势，提升贸易摩擦应对能力水平。加大稳外资力度，落实“外资十条”，提高外资利用水平和成效。更加注重国内市场拓展，组织开展各类招商推介会，大力开拓中西部地区庞大市场，全面优化贸易结构。大力发挥侨乡优势，打侨牌、凝侨心、聚侨力，加大与国际潮籍博士联合会等组织的交流合作，实施“乡贤回归”工程，深入开展“潮文化海外行”，办好“潮州节”等活动。密切与港澳地区的联系往来，深化与泰国曼谷和法国巴黎十三区等国际友城的交流合作。

在更深层次上推进改革。推动机构改革落实落地，着力构建科学规范、运行高效的政务系统。深化商事登记制度改革，实施“多证合一、一照一码”，全面推开“证照分离”“照后减证”改革。持续深入推进“放管服”改革，加大简政放权、减证便民力度，推行“双随机一公开”、跨部门联合监管。推进信用体系建设，强化信用监管。深化人才发展体制机制改革，落实“高层次人才引进办法”，健全完善重才、引才、留才、育才、聚才制度体系，实施“靶向引才”和交流挂任机制，精准引进领军型人才。加快推进“优才卡”项目，建设人才服务机构。推进“僵尸企业”出清，加快国有企业战略重组，整合优化国有资产。推进住房保障货币化改革，完善住房租赁支持政策，加快住房租赁平台建设。深化金融监管体制改革，完善金融风险预警防控机制，强化属地风险处置责任。完成农信社改制并组建农商行，完善金融服务实体经济政策措施。规范政府举债融资，防范政府隐性债务，确保不发生系统性债务风险。深化市级预算编制执行监督管理改革，推行“大专项+任务清单”管理模式。加快完善现代财政制度，对因公出国（境）、公务接待、公务用车、会议、培训、差旅等一般性支出，一律按照不低于5%的幅度压减，提高财政资金配置效率和使用效益。

三、提高发展平衡性和协调性，开创区域城乡发展新格局

围绕“一体两翼三带四平台”建设，不断提升基础设施和公共服务供给质量，力促区域城乡统筹协调发展。

深入实施乡村振兴战略。加大“三农”工作财政投入，推动“六个抓”落实落细，全域推进农村人居环境整治，坚持科学规划、因地制宜，最大限度保护好原生态、原风貌，努力建设生态宜居美丽乡村。全面完成村庄“三清理三拆除三整治”任务，实施“千村示范、万村整治”工程，70%以上的行政村达到干净整洁村标准。大力推进农村危房改造和村道硬化、集中供水、雨污分流、垃圾处理等设施建设,加快农村“厕所革命”。把乡村振兴与精准扶贫结合起来，扎实推进“万企帮万村”“一村一品、一镇一业”“潮州菜师傅”工程，大力发展优势特色扶贫产业，抓实特殊贫困群体脱贫攻坚工作，坚决打好打赢脱贫攻坚战。深化农业供给侧结构性改革，推广“龙头企业+基地+农户”三级联动模式，建立市场化利益联结机制，促进农村一二三产业融合发展。在保持好绿水青山底色的基础上，统筹推动农业现代化、产业化、特色化发展。加快饶平县现代农业（茶叶）产业园建设，争取更多省级产业园落地。稳步推进农村集体产权制度改革。着力提升地方储备粮管理水平。严格落实耕地占补平衡制度，大力推进垦造水田和高标基本农田建设。

全面加快新型城镇化建设。推进国家和省新型城镇化综合试点建设，加快特色小镇创建培育工作。推进中心城区控制性详细规划修编，实现中心城区控规全覆盖。进一步完善土地征储机制，把握政策机遇，强化土地供给保障。加大市政设施建设力度，加快新一轮“八个十”工程建设。推进城管执法体制改革，完善环卫市场化体制，网格化、精细化推进“治六乱”工作。坚持“开前门”与“堵后门”并举，严格落实“双查”机制，铁腕重拳整治土地“三未”“两违”问题，提升节约集约用地水平。加快“最后一公里”水电气路邮建设，推进14个电力主网项目工程建设。

加快推进基础设施建设。突出抓好交通建设，按照“完善骨干网、织密支线网、提升主干道”的思路，实施年度总投资57.1亿元的交通重点项目，重点抓好“一路一港两桥三铁四高五项节点”建设，着力打造“江海陆空、互联互通”的立体综合交通体系。推进“四好农村路”建设，重点实施总里程约100公里的22个先行路和示范路项目。抓好潮州港建设，加快潮州港公用航道一期工程等项目。积极推进外环大桥、东凤大桥等桥梁项目。加快潮汕站站改扩容、梅汕客专建设，争取汕漳沿海高铁在凤泉湖高新区规划设立潮州东站。加快潮汕环线、大潮高速、宁莞高速潮州东联络线等项目建设和潮梅高速前期工作。推进省道S231线、S335线等路段改建和景观提升。突出抓好水利建设，加快韩江新城防洪综合整治工程（一期）、引韩济饶供水工程、粤东灌区续建配套与节水改造工程、省级水利示范县项目和中小河流治理工程（二期）。开工建设北关干渠综合整治提升工程、韩江金山大桥湿地公园提升工程，推进韩江南堤综合整治工程，加快古枫涝区综合整治工程等项目前期工作。

坚决打好污染防治攻坚战。全面深化生态文明建设，针对突出问题，打好重点战役，统筹山水林田湖草系统治理。科学划定“三区三线”，加强生态修复和建设。集中力量打好大气、水、土壤污染防治攻坚战，突出抓好水污染治理，全面落实河长制、湖长制，深入开展饮用水水源地环境问题整治，推进三江水系连通工程潮州项目建设。铁腕推进中央、省环保督察反馈意见整改落实，突出抓好枫江、黄冈河、内洋南总干、内洋西总干等重点流域整治，加快生活污水处理设施及配套管网建设。狠抓市区环保发电厂、饶平宝斗石生活垃圾资源化工程建设和镇级垃圾填埋场整治，实现建制镇污水处理设施全覆盖。系统推进规模化畜禽养殖场污染治理。深入实施环保综合执法机制，严厉打击偷排漏排、超标排放行为。推进国家海洋督查反馈意见整改，加强海岸线修复与保护，严肃查处违法围填海和破坏占用岸线违法行为。全面加快“国家森林城市”创建，实施新一轮绿化潮州大行动，完成森林碳汇造林1.2万亩，森林抚育7.4万亩，新增绿化美化乡村20个。

四、切实保障和改善民生，共建幸福美好新生活

坚持以人民为中心的发展理念，着力提升社会保障水平，优化公共服务供给，继续办好十件民生实事，更好满足人民日益增长的美好生活需要。

扎实推进社会保障。大力提高创业就业服务水平，建设人力资源服务产业园，完善创业担保贷款机制，创建省级创业孵化示范基地，落实“促进就业9条”，新增城镇就业1.5万人。推进社保扩面征缴，落实企业职工养老保险省级统筹制度，推进机关事业单位养老保险制度改革。构建多层次养老服务体系，推进市社会福利院社会化改革和特困人员供养服务机构社会化改革试点工作。提高医疗保险待遇，落实异地联网结算，实现基本医保和大病保险“一站式”实时结算。完善城乡最低生活保障制度，强化低保、特困人员、流浪乞讨人员保障和救助工作，健全社会福利、慈善救济机制。

统筹推进社会事业。加快教育现代化先进市创建步伐，推进十个以上学校建设，探索推进集团化办学模式，全面提升义务教育优质均衡发展水平。扩大学前教育学位供给，大力发展职业教育，不断完善教育体系。突出潮州文化特色，推进市县镇村四级文化体育设施建设。加大非物质文化遗产传承保护，完善非遗数据库建设，不断发展壮大传承人队伍。启动海上丝绸之路申遗工作，完成申遗点保护规划编制。加强文物遗产保育活化工作，鼓励社会力量和民间资本参与文物保护修复。继续深化医药卫生体制改革，加快市中心医院新院、市妇幼保健院改扩建、饶平县人民医院、潮安区妇幼保健院等十个以上医院建设，抓好重大传染病防控，加强基层医疗卫生服务基础设施和人才队伍建设。大力发展“互联网+医疗健康”，优化家庭医生签约服务，完善多层次医疗保障体系建设。做实做大家政服务业，把家政服务办成爱心工程。加大财政民生领域投入力度，提升民生保障能力水平。

创新推进社会治理。创新基层治理模式，推广基层治理职能综合化、服务网格化、平台智能化、治理社会化“四化”建设试点经验。加快“平安视频三期”“雪亮工程”和数字化城市管理平台建设，推动平台对接整合和资源共享，全面提高社会治理的信息化、智能化水平。推进平安潮州建设，加快建设市公安培训基地，深入开展扫黑除恶专项斗争，严厉打击黄赌毒、涉枪涉爆、盗采国家资源、电信网络诈骗、制假贩假等违法犯罪行为，推进全国禁毒示范城市创建，推动“反邪教”“扫黄打非”进基层。加强预防和化解社会矛盾机制建设，坚决维护社会和谐稳定。严格落实安全生产责任制，深化“一岗双责”“三个抓”，深入排查治理风险隐患，深化“城中村”、古城区等高风险区域消防安全整治，加快推进市消防训练基地和消防站建设，坚决防范遏制重特大事故发生。健全公共安全应急管理体系，提升防灾减灾能力。加强食品药品安全监管，确保人民群众生命健康安全。

与此同时，积极推动军民融合发展，加强双拥优抚安置工作，大力支持国防和军队建设。支持工会、共青团、妇联等人民团体工作，进一步做好民族宗教、广播电视、新闻出版、对台、人防、海防、气象、统计、物价、档案、地方志、残疾人、红十字等工作。

五、全面加强党的建设，提升政务服务新水平

认真贯彻落实新时代党的建设总要求，以全面从严治党促进政府治理体系和治理能力现代化。

抓好党的政治建设。全面贯彻习近平新时代中国特色社会主义思想和党的十九大、十九届历次全会精神，深入贯彻落实习近平总书记对广东重要讲话和重要指示精神，牢固树立“四个意识”，坚定“四个自信”，践行“两个维护”，始终在思想上政治上行动上同以习近平同志为核心的党中央保持高度一致。严明政治纪律和政治规矩，确保政令畅通、令行禁止。健全大督查工作机制，严格责任落实，严抓跟踪督查，力促各项决策部署不折不扣落地见效。

抓好民主法治建设。认真执行人大及其常委会各项决议决定，及时办理人大代表建议和政协提案，自觉接受人大、政协和社会各界监督。拓宽重大决策公众参与平台，广泛听取社会各方面意见和建议。抓好“七五”普法，强化法治思维，完善立法工作机制，推进重点领域立法。深化行政执法体制改革，加强重大行政决策合法性审查，推动法治政府建设再上新台阶。

抓好政务服务建设。加快“数字政府”建设，推进政务服务“一网通办”和“互联网+政务服务”、审批服务便民化，建设一体化在线政务服务平台。推动全市政务信息系统互联和公共数据共享，建立大数据驱动的政务信息化服务模式。进一步完善政务服务体系，提升镇（街）政务服务中心管理和运作水平，优化村（居）公共服务站服务功能。

抓好廉洁政府建设。严格落实党风廉政建设责任制，加强廉政教育，提升干部拒腐防变思想认识，防范商品交换原则向党内渗透，规范政商交往行为，加快构建亲清新型政商关系。贯彻落实中央八项规定及其实施细则精神，驰而不息纠正“四风”，营造风清气正政治生态。厉行勤俭节约，缩减“三公”经费，严控一般性支出。加强审计监督，确保权力规范运行。

各位代表，让我们紧密团结在以习近平同志为核心的党中央周围，高举习近平新时代中国特色社会主义思想伟大旗帜，深入学习贯彻党的十九大精神，深入贯彻落实习近平总书记对广东重要讲话和重要指示精神，在省委、省政府和市委的坚强领导下，全面推动思想再解放、改革再深入、工作再落实，开拓创新，锐意进取，为建设沿海经济带上的特色精品城市而努力奋斗！
